# Dengue and Other Arbovirus Infections among Schoolchildren, Haiti, 2021

**DOI:** 10.3201/eid3102.240791

**Published:** 2025-02

**Authors:** Rigan Louis, Tracey L. Moquin, Carla Mavian, Assonic Barthelemy, Ruiyu Pu, Benjamin Anderson, V. Madsen Beau De Rochars, Maureen T. Long, Marco Salemi, John A. Lednicky, J. Glenn Morris

**Affiliations:** University of Florida College of Nursing, Gainesville, Florida, USA (R. Louis); University of Florida Emerging Pathogens Institute, Gainesville (R. Louis, T.L. Moquin, C. Mavian, R. Pu, B. Anderson, V.M.B. De Rochars, M.T. Long, M. Salemi, J.A. Lednicky, J.G. Morris, Jr.); University of Florida College of Public Health and Health Professions, Gainesville (T.L. Moquin, B. Anderson, V.M.B. De Rochars, J A. Lednicky); Smithsonian's National Zoo and Conservation Biology Institute, Washington, DC, USA (C. Mavian); University of Florida College of Medicine, Gainesville (C. Mavian, M. Salemi); Love A Child Foundation Medical Clinic, Fond Parisien, Haiti (A. Barthelemy)

**Keywords:** dengue, dengue virus, arboviruses, viruses, dengue type 2, chikungunya, Zika virus, Haiti

## Abstract

In 2021, we screened 91 children in Haiti with acute undifferentiated febrile illness for arbovirus infections. We identified a major outbreak of dengue virus type 2, with 67% of the children testing positive. Two others were positive for chikungunya East/Central/South African IIa subclade, and 2 were positive for Zika virus.

Since 2010, dengue (DENV), chikungunya (CHIKV), and Zika (ZIKV) viruses have been responsible for major outbreaks in the Caribbean region (*1*,*2*). In Haiti, reported DENV cases have tended to assume an endemic/sporadic pattern; cases surged in 2020–2022. CHIKV was responsible for epidemic disease in Haiti in 2014; ZIKV was associated with outbreaks in 2014–2016. Haiti has reported no CHIKV or ZIKV infections since 2019 (*3*,*4*). CHIKV and ZIKV are known to circulate intermittently in Brazil and other parts of South America and Central America since 2019 (*3*,*4*). Given the limitations of formal surveillance systems and arbovirus diagnostic capabilities in Haiti, we assessed the status of those viruses in Haiti to determine possible origins or evidence of persistence of viruses that might be present. We established surveillance during February–December 2021 for arboviral diseases among children attending schools operated by the Love a Child (LAC) Foundation (Fond Parisien, Haiti).

LAC schoolchildren who were brought to the LAC medical clinic for care for subjective fever without an obvious source of infection were invited to participate in the study; 91 children with febrile illness were enrolled. We collected clinical and epidemiologic data from participants and their parents and serum, nasal swab, and urine samples from case-patients, in accordance with standard protocols (*5*–*8*) (Appendix 1 Tables 1, 2). We screened serum samples by reverse transcription PCR (RT-PCR) for DENV, CHIKV, ZIKV, and Mayaro virus (MAYV) (*5*–*8*). Because COVID-19 was circulating in the community at the time of the study (*9*), we screened nasal swab samples from patients by RT-PCR for SARS-CoV-2. We further sequenced a random subset of 6 DENV-2–positive samples and 2 DENV-2–positive samples from patients positive for SARS-CoV-2, together with all samples positive for CHIKV, ZIKV, and SARS-CoV-2, and submitted sequences to GenBank/GISAID (Appendix 1 Table 1).

During the surveillance period, we identified a major DENV-2 outbreak (Figure 1); a total of 61 (67%) of the 91 participants had a positive serum RT-PCR result for DENV-2 virus RNA. Clinical findings were not significantly different among children who were DENV-2–positive than among those who were febrile but DENV-2–negative (Appendix 1 Table 2), underscoring the difficulties inherent in making a diagnosis of dengue fever based solely on clinical presentation. Among DENV-2–positive participants, 31% reported using mosquito nets, compared with 13% of DENV-2–negative participants (p = 0.07 by 2-tailed Fisher exact test); although not statistically significant, the trend is the opposite of what we would expect if mosquito nets were an effective tool for prevention of dengue.

**Figure 1:**
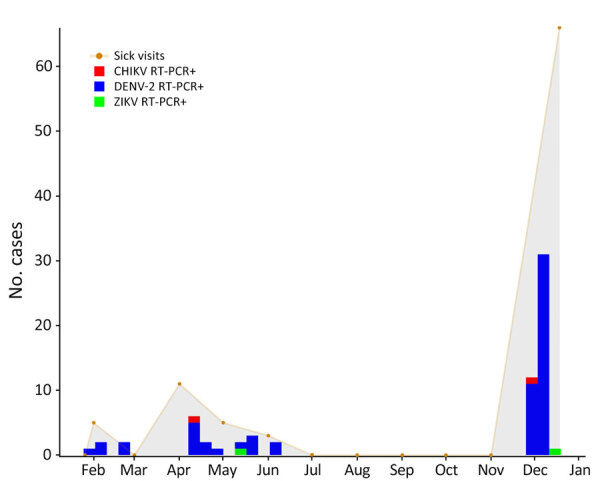
Numbers of children who had acute undifferentiated febrile illness and tested positive by RT- PCR for CHIKV, DENV-2, and ZIKV virus, by month, Haiti, 2021. Gray shading indicates the number of children seen in the clinic with symptoms of fever of undetermined etiology. CHIKV, chikungunya virus; DENV, dengue virus; RT-PCR, reverse transcription PCR; ZIKV, Zika virus; +, positive.

We detected CHIKV vRNA by RT-PCR in serum samples from 2 children (Appendix 1 Table 1). In contrast to the 2014 CHIKV epidemic in Haiti, infections were caused by a strain within the East/Central/South African (ECSA) IIa American subclade (*7*,*10*) (Appendix 1 Table 2). Given that we were finding active ECSA CHIKV cases by RT-PCR, we were interested in assessing the frequency with which CHIKV antibodies were present among children in our cohort. IgG seropositivity was highly associated with age (Figure 2): of the 45 children <7 years of age, 2% were seropositive for CHIKV IgG, versus 78% of children >7 years of age (p<0.001 by 2-tailed Fisher exact test). Ten children were CHIKV IgM positive but CHIKV negative on RT-PCR. Eight of those 10 children, all >7 years of age, were also IgG positive; 7/8 were positive by RT-PCR for DENV-2.

**Figure 2:**
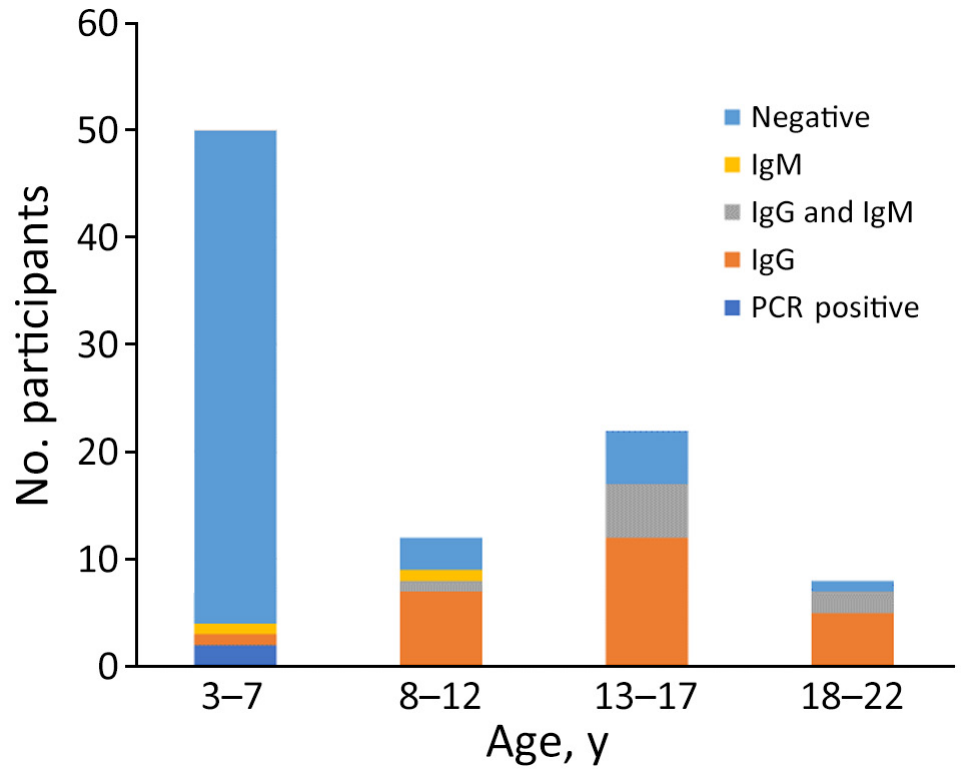
Results of chikungunya serologic testing by age group in study of arboviruses in children, Haiti.

We detected ZIKV vRNA in serum samples from 2 children, 16 and 7 years of age (Appendix 1 Table 1). Both had subjective fever; the 16-year-old reported headache, arthralgias, and upper respiratory symptoms, whereas the 7-year-old noted some abdominal pain but had no other complaints. Neither had a rash. Two children (both 3 years of age) were positive for SARS-CoV-2 by nasal swab; both also were serum RT-PCR positive for DENV-2 (Appendix 1 Table 1). All RT-PCR test results for MAYV were negative.

Phylogenetic analysis revealed 2 DENV-2 clades identified in Haiti and designated as American/Asian genotype—Haiti clade 1 and 2 (Figure 3; Appendix 1; Appendix 2 Table). The DENV-2 strains from our study were clade 2, a Dominican Republic strain that initially seeded Haiti and returned in March 2020 (95% highest posterior density [HPD] June 2019–December 2020) (Figure 4). Although we could not infer directionality of spread, our analyses indicate continuous flow of DENV-2 between the 2 countries, leading to independent introductions into both.

**Figure 3:**
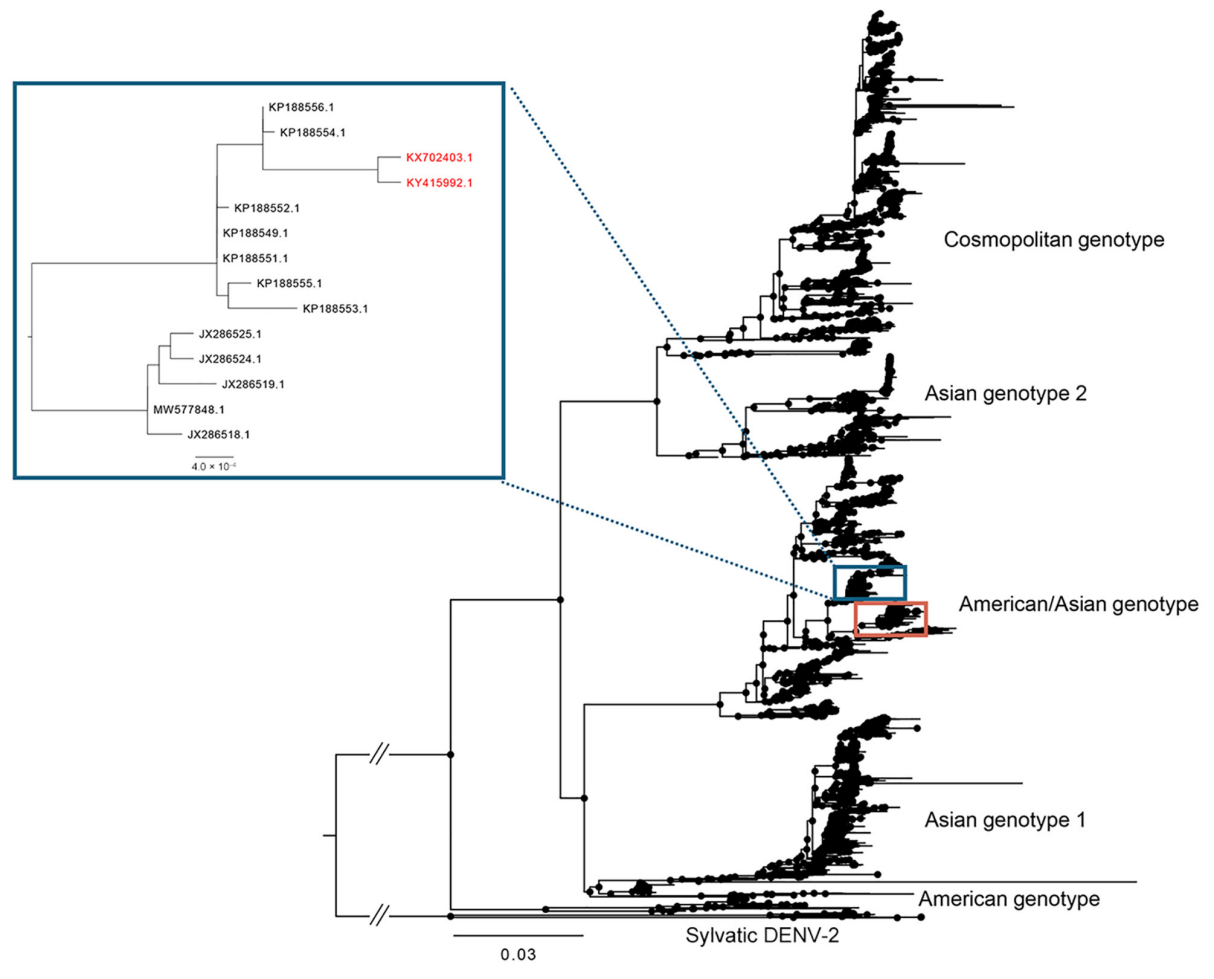
Global maximum-likelihood phylogeny of DENV-2 genotypes for all DENV-2 isolates available in GenBank inferred using IQ-TREE version 2.3.2 (http://www.iqtree.org). Haiti sequences cluster in 2 distinct clades (in boxes). Inset shows location of Haiti clade 1 strains KY415992, isolated in 2014, and Haiti strain KX702403, collected in 2016 (red text). Branch lengths reflect genetic distances. Black circles at each node shows strong statistical support based on ultrafast posterior probability bootstrap support (>90%). Scale bar represents nucleotide substitutions per site. DENV, dengue virus.

**Figure 4:**
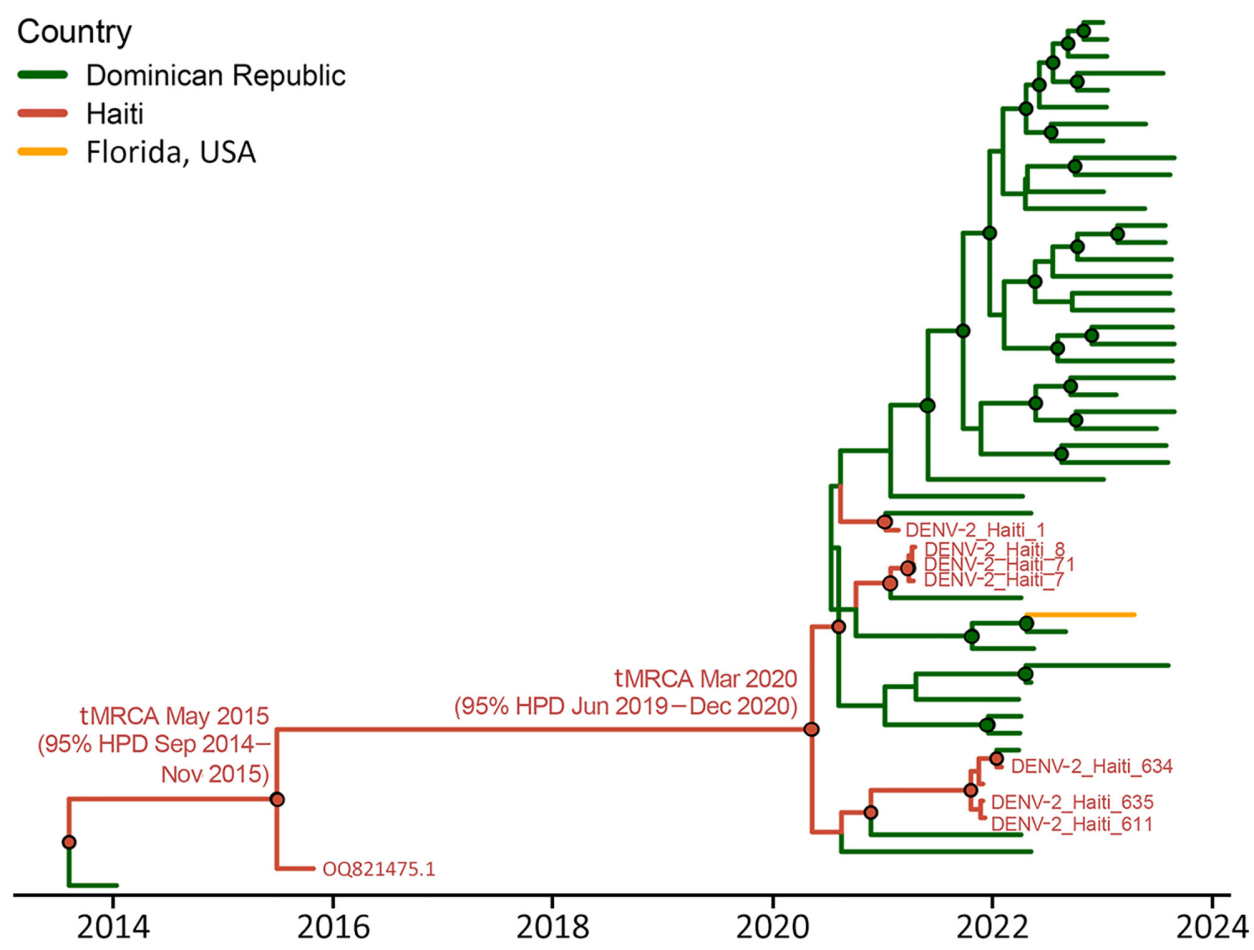
Phylogeography for the American/Asian genotype of DENV containing new Haiti isolates. Time-scaled phylogenetic maximum clade creditability tree for Haiti clade 2 (including GenBank accession no. OQ821475.1, isolated in 2015, and new 2021 strains, shown in red) was inferred using the phylogeographic frameworks in BEAST version 1.10.4 (https://beast.community) and enforcing the Bayesian Skyline demographic prior with an uncorrelated lognormal relaxed clock. Branch colors represent country of origin of the genome, and posterior probability bootstrap support >0.90 at each node is shown with a circle colored by the ancestral country of origin. DENV, dengue virus; HPD, highest posterior density; tMRCA, time to most recent common ancestor.

CHIKV-positive samples from this study, together with previously reported 2016 CHIKV mosquito isolates (*7*), cluster with strains belonging to the ECSA IIa Brazil-Haiti subclade, isolated in Brazil in 2014 (Appendix 1 Figure 2) (*2*,*10*,*11*). Including the 2016 mosquito isolates, the estimated time to the most recent common ancestor was November 2015 (95% HPD October 2014–April 2016) (Appendix 1 Figure 3). Sequences of both ZIKV strains were in what was previously designated as Zika Haiti clade 1, a Haiti-Brazil lineage found in Haiti in 2014 (Appendix 1 Figure 4). Time to most recent common ancestor for the emergence of the new strains is estimated at December 2020 (95% HPD August 2018–January 2021), suggesting that ZIKV has continued to circulate in Haiti since 2014 (95% HPD October 2013–August 2014) (Appendix 1 Figure 5) (*6**,**12*). 

The generalizability of our study is limited by the small sample size and the collection of all samples from a single geographic location; nonetheless, it provides a snapshot of arboviruses circulating in Haiti in 2021. We unexpectedly identified a previously unrecognized dengue type 2 outbreak among children at the LAC school, in keeping with reports from the Ministere de la Sante Publique et de la Population of a surge of DENV infections nationally (*13*). Work by our group and others supports the concept that DENV is endemic in Haiti. At various times, Ministere de la Sante Publique et de la Population has reported occurrence of all 4 dengue serotypes (*13*). Given that occurrence of severe dengue has been associated with serial infection with different DENV serotypes, one might have expected an increased likelihood of severe dengue cases in Haiti (*14*). However, severe dengue has been rarely reported from Haiti (*13*,*14*), and, in our experience, is uncommon among children (A. Barthelemy, unpub. data).

The sharp increase in CHIKV IgG seropositivity after age 7 we observed is consistent with infection of virtually the entire population during the initial 2014 Asian clade epidemic; children born after the 2014 epidemic appear to have had minimal exposure to the virus, as reflected in the negative IgG results. Our phylodynamic studies are consistent with introduction of the Brazil-Haiti CSA IIa CHIKV strain into Haiti around 2015, shortly after its identification in Brazil (*2*). What is somewhat unexpected is that this same ECSA CHIKV strain has persisted in Haiti since 2015 or earlier. The 2016 ECSA IIa strain was isolated from *Aedes albopictus* mosquitoes; we isolated the Asia clade from *Ae. aegypti* mosquitoes (*7*), highlighting the potential for ECSA CHIKV transmission in areas in which *Ae. albopictus* mosquitoes are present, but not *Ae. aegypti* mosquitoes. The apparently low-level persistence of ZIKV in Haiti is also of interest; the strain we initially isolated in 2014 was still present in 2021 in this study (*6*,*12*).

Warming temperatures through the Caribbean and other parts of the Americas could cause further spread of arbovirus species and increased arbovirus transmission (*15*); rapid spread of DENV has been documented (*13*). Our findings that both CHIKV and ZIKV were present but unrecognized in Haiti, coupled with limited availability of specific diagnostics, raise concern that arboviruses may spread unrecognized into other areas in the region.

Appendix 1 Additional information about dengue and other arbovirus infections among schoolchildren, Haiti, 2021.

Appendix 2 List of strains included in phylogenetic analyses of dengue and other arboviruses among schoolchildren, Haiti, 2021.
