# Supplementary material for: Dengue and Other Arbovirus Infections among Schoolchildren, Haiti, 2021
Source: Emerg Infect Dis. 2025 Feb;31(2):336–40. doi: 10.3201/eid3102.240791 (PMC11845146; doi:10.3201/eid3102.240791)
Supplement: Appendix 1 — Additional information about dengue and other arbovirus infections among schoolchildren, Haiti, 2021. [file 24-0791-Techapp-s1.pdf]

Article DOI: https://doi.org/10.3201/eid3102.240791

EID cannot ensure accessibility for supplementary materials supplied by authors. Readers who have difficulty accessing supplementary content should contact the authors for assistance.

Dengue and Other Arbovirus Infections among Schoolchildren, Haiti, 2021

Appendix 1

Supplemental Methods/Results

In keeping with previously described methods (*1*), between February and December 2021, we enrolled 91 children attending schools operated by the Love a Child (LAC) Foundation in Fond Parisien, Haiti, who presented to the LAC medical clinic with reports of subjective fever without an obvious source of infection. The study was approved by the University of Florida IRB and the Haitian National IRB; signed parental informed consent was obtained for all participants, with patient assent. Clinical and demographic data were collected from participants and their parents, and samples of serum, a nasal swab, and urine were collected from case patients. Details of enrollment and sample collection have been previously described in studies from our group (*1*–*6*).

As COVID was circulating in Haiti during the study period (*7*), nasal swabs from participants were screened for SARS-CoV-2 by RT-PCR (*8*). RT-PCR was used to screen serum samples for viral RNA (vRNA) from chikungunya (CHIKV), Zika (ZIKV), dengue (DENV), and/or mayaro (MAYV) viruses. We initially screened for DENV vRNA by using the primer system of Santiago et al. (*9*), followed by the primer system of Huhtamo et al. (*10*) since we have found that some contemporary dengue virus strains from Haiti are ineffectively detected using the Santiago primers. The extracted vRNAs were tested for CHIKV based on methods described by Johnson et al. (*11*) to detect both Asian an East/Central/South African genotypes of CHIKV (*2*). ZIKV vRNA was detected by using primers described by Lanciotti et al. (*12*). Screening for MAYV was done by using the nested primer systems described by Bronzoni et al. (*13*).

SARS-CoV-2 was sequenced as previously described (*7*). In brief, Sanger sequencing using a genome walking (tiling) approach and 5′ and 3′ RACE was performed as described for CHIKV (*14*), Dengue virus (*15*), and ZIKV (*3*,*16*). Where possible, the 5′ and 3′ ends of the virus genomes were determined using a RACE (rapid amplification of cDNA ends) kit (5′ RACE System for Rapid Amplification of cDNA ends, version 2.0), ThermoFisher Scientific, Waltham, MA) following the manufacturer's instructions. Furthermore, flavivirus RNAs were polyadenylated before 3′ RACE using a Poly(A) Tailing kit (Invitrogen, ThermoFisher Scientific). GenBank or GISAID accession numbers for sequences are included in Appendix 1 Table 1.

Serum samples were screened for CHIKV IgG and IgM antibodies by using a commercial ELISA (CHIKjj Detect IgM-capture ELISA kit and CHIKjj DetectTM IgG ELISA Kit Inbios International, Inc., Seattle, USA) according to the manufacturer's directions (*17*). With respect to the commercial kit used in this study, a sensitivity and specificity of 98.6% and 92.9% has been reported (*18*). We identified two acutely ill children with a positive CHIKV RT-PCR and negative IgM and IgG titers, consistent with early CHIKV infection (*19*,*20*). We also identified two children with negative CHIKV RT-PCR but a positive IgM and a negative IgG response; this would be consistent with patients later in the course of illness, at a point where IgM had reduced viral titers but IgG was not yet detectable. Eight other children (all greater than 7 years of age) had negative CHIKV RT-PCR but positive IgM and IgG; seven of these were also DENV2-positive. In general, CHIKV antibody is detectable at the end of viremia, with IgM detectable as early as 2 to 4 days after infection (*19–21*). While IgM antibody can persist post-exposure in some patients, it tends to be test and location dependent: in some studies IgM persisted up to 12 months in less than 15% of patients (*22–24*) while in a Cameroon study this percentage was greater than 50% (*25*). We cannot exclude the possibility that the children with a positive IgM for CHIKV represented acute CHIKV infections, but there is also a reasonable likelihood that this reflects prior exposure to CHIKV with a positive IgG and persistently positive IgM. Regardless of the actual case number, our data provide strong evidence that acute ECSA-clade CHIKV infections were occurring among members of our cohort in April and again in December 2021.

Phylogenetic analysis

To assess the relatedness of the CHIKV, ZIKV, and DENV strains identified to earlier isolates from Haiti and other geographic locations, publicly available genomes were obtained from NCBI (DENV n = 3,816; CHIKV n = 1,032; ZIKV n = 574; Appendix 2 Table), aligned with MAFFT version 7.520 (*26*), and used to infer a maximum likelihood phylogeny with IQ-TREE version 2.3.2 (*27*). Bayesian phylogeography ancestral state reconstruction (*28*) of the monophyletic clade containing the Haitian genomes was run using BEAST version 1.10.4 (*29*,*30*) and a relaxed clock and Bayesian Skyline plot as demographic prior (*31*) and an asymmetric substitution model for discrete traits (i.e., locations). Root-to-tip analyses are shown in Appendix 1 Figure 1. we assessed possible recombination for our new CHIKV, DENV2 and ZIKV genomes using RDP5 (*32*) and analyses did not show evidence of recombination.

Two DENV2 clades were identified in Haiti, designated as American/Asian genotype – Haiti clade 1 and 2. The DENV2 strains from the current study cluster in the American/Asian genotype – Haiti clade 2 that contains DENV2 strains from the DR and Florida (Figures 3, 4). The structure of this clade is quite intermixed, with a DR strain initially seeding Haiti with subsequent reseeding of the DR in March 2020 (95% HPD June 2019–December 2020). A long branch, indicating a gap in sampling, is connecting the first Haitian strain OQ821475.1 isolated in 2015 to the origin of DR and later Haitian outbreaks. Within the clade containing recent DR and Haitian strains, Haitian strains contributed only in part to introductions into the DR, as only a few DR strains are directly branching off Haitian ones. Although the gap in samples in Haiti between 2016–2021 does not allow us to infer directionality of spread, our analyses clearly indicate continuous flow of DENV-2 between the two countries leading to independent introductions into Haiti and DR.

For CHIKV, the time-scaled Maximum Clade Credibility (MCC) phylogeny of the ECSA lineage (Appendix 1 Figure 2) was inferred using the Bayesian Skyline demographic enforcing an uncorrelated lognormal relaxed clock as determined by model testing. While the 2014 CHIKV epidemic in the Americas aligned with the Asian lineage, our MCC phylogeny showed (in accordance with the ML phylogeny) that the 2021 clinical CHIKV genomes obtained as part of this study, together with our previously reported 2016 CHIKV mosquito isolates (*3*), cluster with strains isolated in Brazil in 2014 belonging to the ECSA IIa Brazil-Haiti subclade (*3*,*33*–*36*). The 2021 clinical CHIKV strains shared a common ancestor with previously circulating Haitian

CHIKV dating to September 2020 (95% highest posterior density [HPD] interval of October 2019-April 2021). Including our 2016 mosquito isolates, the estimated time to the most recent common ancestor (TMRCA) was November 2015 (95% HPD October 2014-April 2016) (Appendix 1 Figure 3).

For ZIKV (Appendix 1 Figure 4) our two clinical strains were in what we had previously designated as Zika Haiti clade 1, a Haiti-Brazil lineage with new strains clustering near the KU509998.3 strain that was found in Haiti in 2014. The TMRCA for the emergence of the new strains is estimated at December 2020 (95%HPD August 2018–January 2021), suggesting that circulation of ZIKV was maintained in Haiti since the introduction of KU509998.3 in August 2014 (HPD 95% October 2013–August 2014)(Appendix 1, Figure 5 (*2*,*17*). Brazilian strains branch off the KU509998.3 strain indicating introduction of ZIKV in Brazil from Haiti, and back to Haiti in 2016 when the strain MF783073.1 was isolated.

References

1. Ball JD, Elbadry MA, Telisma T, White SK, Chavannes S, Anilis MG, et al. Clinical and epidemiologic patterns of chikungunya virus infection and coincident arboviral disease in a school cohort in Haiti, 2014–2015. Clin Infect Dis. 2019;68:919–26. PubMed https://doi.org/10.1093/cid/ciy582

2. White SK, Mavian C, Salemi M, Morris JG Jr, Elbadry MA, Okech BA, et al. A new "American" subgroup of African-lineage Chikungunya virus detected in and isolated from mosquitoes collected in Haiti, 2016. PLoS One. 2018;13:e0196857. PubMed https://doi.org/10.1371/journal.pone.0196857

3. Lednicky J, Beau De Rochars VM, El Badry M, Loeb J, Telisma T, Chavannes S, et al. Zika Virus outbreak in Haiti in 2014: molecular and clinical data. PLoS Negl Trop Dis. 2016;10:e0004687. PubMed https://doi.org/10.1371/journal.pntd.0004687

4. Blohm G, Elbadry MA, Mavian C, Stephenson C, Loeb J, White S, et al. Mayaro as a Caribbean traveler: evidence for multiple introductions and transmission of the virus into Haiti. Int J Infect Dis. 2019;87:151–3. PubMed https://doi.org/10.1016/j.ijid.2019.07.031

5. Lednicky JA, White SK, Mavian CN, El Badry MA, Telisma T, Salemi M, et al. Emergence of *Madariaga virus* as a cause of acute febrile illness in children, Haiti, 2015–2016. PLoS Negl Trop Dis. 2019;13:e0006972. PubMed https://doi.org/10.1371/journal.pntd.0006972

6. Elbadry MA, Durães-Carvalho R, Blohm GM, Stephenson CJ, Loeb JC, White SK, et al. Orthobunyaviruses in the Caribbean: Melao and Oropouche virus infections in school children in Haiti in 2014. PLoS Negl Trop Dis. 2021;15:e0009494. PubMed https://doi.org/10.1371/journal.pntd.0009494

7. Tagliamonte MS, Mavian C, Zainabadi K, Cash MN, Lednicky JA, Magalis BR, et al. Rapid emergence and spread of severe acute respiratory syndrome coronavirus 2 gamma (P.1) variant in Haiti. Clin Infect Dis. PubMed https://doi.org/10.1093/cid/ciab736

8. US Food and Drug Administration. Real-time RT-PCR diagnostic panel approved by FDA for emergency use only. [cited 2024 Oct 14]. https://www.fda.gov/media/134922/download

9. Santiago GA, Vergne E, Quiles Y, Cosme J, Vazquez J, Medina JF, et al. Analytical and clinical performance of the CDC real time RT-PCR assay for detection and typing of dengue virus. PLoS Negl Trop Dis. 2013;7:e2311. PubMed https://doi.org/10.1371/journal.pntd.0002311

10. Huhtamo E, Hasu E, Uzcátegui NY, Erra E, Nikkari S, Kantele A, et al. Early diagnosis of dengue in travelers: comparison of a novel real-time RT-PCR, NS1 antigen detection and serology. J Clin Virol. 2010;47:49–53. PubMed https://doi.org/10.1016/j.jcv.2009.11.001

11. Johnson BW, Russell BJ, Goodman CH. Laboratory diagnosis of chikungunya virus infections and commercial sources for diagnostic assays. J Infect Dis. 2016;214(suppl 5):S471–4. PubMed https://doi.org/10.1093/infdis/jiw274

12. Lanciotti RS, Kosoy OL, Laven JJ, Velez JO, Lambert AJ, Johnson AJ, et al. Genetic and serologic properties of Zika virus associated with an epidemic, Yap State, Micronesia, 2007. Emerg Infect Dis. 2008;14:1232–9. PubMed https://doi.org/10.3201/eid1408.080287

13. Bronzoni RV, Moreli ML, Cruz AC, Figueiredo LT. Multiplex nested PCR for Brazilian Alphavirus diagnosis. Trans R Soc Trop Med Hyg. 2004;98:456–61. PubMed https://doi.org/10.1016/j.trstmh.2003.09.002

14. Cherabuddi K, Iovine NM, Shah K, White SK, Paisie T, Salemi M, et al. Zika and chikungunya virus co-infection in a traveller returning from Colombia, 2016: virus isolation and genetic analysis. JMM Case Rep. 2016;3:e005072. PubMed https://doi.org/10.1099/jmmcr.0.005072

15. White S, KIovine NM, Nickels LC, Morris JG, Lednicky JA. Complete genome sequence of dengue virus type 2 from a resident of north-central Florida with locally transmitted dengue fever. Genome Announc. 2017;5:10.1128/genomea.00782-17. https://doi.org/10.1128/genomea.00782-17

16. Alam MM, Mavian C, Okech BA, White SK, Stephenson CJ, Elbadry MA, et al. Analysis of Zika virus sequence data associated with a school cohort in Haiti. Am J Trop Med Hyg. 2022;107:873–80. PubMed https://doi.org/10.4269/ajtmh.22-0204

17. Kikuti M, Tauro LB, Moreira PSS, Nascimento LCJ, Portilho MM, Soares GC, et al. Evaluation of two commercially available chikungunya virus IgM enzyme-linked immunoassays (ELISA) in a setting of concomitant transmission of chikungunya, dengue and Zika viruses. Int J Infect Dis. 2020;91:38–43. PubMed https://doi.org/10.1016/j.ijid.2019.11.001

18. Andrew A, Navien TN, Yeoh TS, Citartan M, Mangantig E, Sum MSH, et al. Diagnostic accuracy of serological tests for the diagnosis of chikungunya virus infection: A systematic review and meta-analysis. PLoS Negl Trop Dis. 2022;16:e0010152. PubMed https://doi.org/10.1371/journal.pntd.0010152

19. Johnson BW, Russell BJ, Goodman CH. Laboratory diagnosis of chikungunya virus infections and commercial sources for diagnostic assays. J Infect Dis. 2016;214(suppl 5):S471–4. PubMed https://doi.org/10.1093/infdis/jiw274

20. Kumar R, Shrivastava T, Samal S, Ahmed S, Parray HA. Antibody-based therapeutic interventions: possible strategy to counter chikungunya viral infection. Appl Microbiol Biotechnol. 2020;104:3209–28. PubMed https://doi.org/10.1007/s00253-020-10437-x

21. Grivard P, Le Roux K, Laurent P, Fianu A, Perrau J, Gigan J, et al. Molecular and serological diagnosis of chikungunya virus infection. Pathol Biol (Paris). 2007;55:490–4. PubMed https://doi.org/10.1016/j.patbio.2007.07.002

22. do Nascimento Costa DM, Coelho MRCD, Gouveia PAC, Bezerra LA, Marques CD, Duarte ALBP, et al. Long-term persistence of serum-specific anti-chikungunya IgM antibody—a case study of Brazilian patients. J Brazilian Soc Trop Med. 2021;54:e0855-2020. https://doi.org/10.1590/0037-8682-0855-2020

23. Moro ML, Grilli E, Corvetta A, Silvi G, Angelini R, Mascella F, et al.; Study Group "Infezioni da Chikungunya in Emilia-Romagna." Long-term chikungunya infection clinical manifestations

after an outbreak in Italy: a prognostic cohort study. J Infect. 2012;65:165–72. PubMed https://doi.org/10.1016/j.jinf.2012.04.005

24. Pierro A, Rossini G, Gaibani P, Finarelli AC, Moro ML, Landini MP, et al. Persistence of anti-chikungunya virus-specific antibodies in a cohort of patients followed from the acute phase of infection after the 2007 outbreak in Italy. New Microbes New Infect. 2015;7:23–5. PubMed https://doi.org/10.1016/j.nmni.2015.04.002

25. Demanou M, Antonio-Nkondjio C, Ngapana E, Rousset D, Paupy C, Manuguerra JC, et al. Chikungunya outbreak in a rural area of Western Cameroon in 2006: a retrospective serological and entomological survey. BMC Res Notes. 2010;3:128. PubMed https://doi.org/10.1186/1756-0500-3-128

26. Katoh K, Standley DM. A simple method to control over-alignment in the MAFFT multiple sequence alignment program. Bioinformatics. 2016;32:1933–42. PubMed https://doi.org/10.1093/bioinformatics/btw108

27. Nguyen LT, Schmidt HA, von Haeseler A, Minh BQ. IQ-TREE: a fast and effective stochastic algorithm for estimating maximum-likelihood phylogenies. Mol Biol Evol. 2015;32:268–74. PubMed https://doi.org/10.1093/molbev/msu300

28. Lemey P, Rambaut A, Drummond AJ, Suchard MA. Bayesian phylogeography finds its roots. PLOS Comput Biol. 2009;5:e1000520. PubMed https://doi.org/10.1371/journal.pcbi.1000520

29. Drummond AJ, Rambaut A. BEAST: Bayesian evolutionary analysis by sampling trees. BMC Evol Biol. 2007;7:214. PubMed https://doi.org/10.1186/1471-2148-7-214

30. Hasegawa M, Kishino H, Yano T. Dating of the human-ape splitting by a molecular clock of mitochondrial DNA. J Mol Evol. 1985;22:160–74. PubMed https://doi.org/10.1007/BF02101694

31. Drummond AJ, Rambaut A, Shapiro B, Pybus OG. Bayesian coalescent inference of past population dynamics from molecular sequences. Mol Biol Evol. 2005;22:1185–92. PubMed https://doi.org/10.1093/molbev/msi103

32. Martin DP, Varsani A, Roumagnac P, Botha G, Maslamoney S, Schwab T, et al. RDP5: a computer program for analyzing recombination in, and removing signals of recombination from, nucleotide sequence datasets. Virus Evol. 2021;7:veaa087. PubMed https://doi.org/10.1093/ve/veaa087

33. de Souza WM, Ribeiro GS, de Lima STS, de Jesus R, Moreira FRR, Whittaker C, et al. Chikungunya: a decade of burden in the Americas. Lancet Reg Health Am. 2024;30:100673. PubMed https://doi.org/10.1016/j.lana.2023.100673

34. Nunes MRT, Faria NR, de Vasconcelos JM, Golding N, Kraemer MUG, de Oliveira LF, et al. Emergence and potential for spread of chikungunya virus in Brazil. BMC Med. 2015;13:102. PubMed https://doi.org/10.1186/s12916-015-0348-x

35. Cunha MS, Cruz NVG, Schnellrath LC, Medaglia MLG, Casotto ME, Albano RM, et al. Autochthonous transmission of East/Central/South African genotype chikungunya virus, Brazil. Emerg Infect Dis. 2017;23:1737–9. PubMed https://doi.org/10.3201/eid2310.161855

36. Charlys da Costa A, Thézé J, Komninakis SCV, Sanz-Duro RL, Felinto MRL, Moura LCC, et al. Spread of Chikungunya virus East/Central/South African genotype in northeast Brazil. Emerg Infect Dis. 2017;23:1742–4. PubMed https://doi.org/10.3201/eid2310.170307

Appendix 1 Table 1. Children with positive RT-PCR for CHIKV, ZIKV, DENV and/or SARS-CoV-2, for whom sequence data were available

Child	Age, y	Isolation, month	Source	Virus	GenBank #
1–05	3	April 2021	serum	CHIKV	PP319439
2–38	4	Dec 2021	serum	CHIKV	PP319440
3–15	16	May 2021	serum	ZIKV	PP316139
4–36	7	Dec 2021	serum	ZIKV	PP316140
5–71	4	April 2021	serum	DENV2	PP761426
6–609	3	Dec 2021	serum	DENV2	PP764026
7–611	3	Dec 2021	serum	DENV2	PP764106
			nasal swab	SARS-CoV-2	(GISAID accession # pending)
8–635	3	Dec 2021	serum	DENV2	PP764239
9–634	3	Feb 2021	serum	DENV2	PP764465
			nasal swab	SARS-CoV-2	(GISAID accession # pending)
10–1	14	Feb 2021	serum	DENV2	PP764833
11–7	3	April 2021	serum	DENV2	PP766249
12–8	6	April 2021	serum	DENV2	PP769194

Appendix 1 Table 2. Clinical and epidemiologic characteristics of case patients*

Characteristic	N	Mean	Range	SD	(df)	p value	95% CI
Overall		9		5.74			
DENV PCR+		9	(3–22)	5.59	(90)	.32†	-371 to 1.27
DENV PCR-		10		6.04			
Household size							
Overall	91	6		1.85			
DENV PCR+		5.51	(2–11)	1.55	(60)	.04†	-1.81 to −0.01
DENV PCR-		6.42		2.23			
Use mosquito net							
Overall	23						
DENV PCR+	19					.074	0.89–13.87
DENV PCR-	4						
DENV_PCR+							
Overall	61						
Male	28					.02	0.12–0.94
Female	33						
Symptoms				OR		p value	95%CI
Abdominal pain							
Overall	12						
DENV PCR+	7			0.68		.53	0.17–2.98
DENV PCR-	5						
Fever							
Overall	91						

Characteristic	N	Mean	Range	SD	(df)	p value	95% CI
Headache							
Overall	45						
DENV PCR+	31			1.25		.66	0.48–3.28
DENV PCR-	14						
Myalgia							
Overall	8						
DENV PCR+	5			0.84		1	0.15–5.77
DENV PCR-	3						
Ostealgia							
Overall	10						
DENV PCR+	6			0.74		.73	0.16–3.87
DENV PCR-							
Skin rash/pruritis	4						
Overall	18						
DENV PCR+	11			0.76		.59	0.23–2.61
DENV PCR-	7						
Retro-orbital pain							
Overall	8						
DENV PCR+	6			1.57		,71	0.25–16.92
DENV PCR-	2						
Cough							
Overall	69						
DENV PCR+	43			0.59		.45	0.17–1.82
DENV PCR-	26						
Rhinorrhea							
Overall	47						
DENV PCR+	31			0.97		1	0.37–2.51
DENV PCR-	16						
Vomiting							
Overall	12						
DENV PCR+	7			0.68		.53	0.17–2.98
DENV PCR-	5						

*All statistical analysis were performed using R Studio 2024.04.1.
†Welch T-test; all the other statistics were obtained with Fisher exact tests.

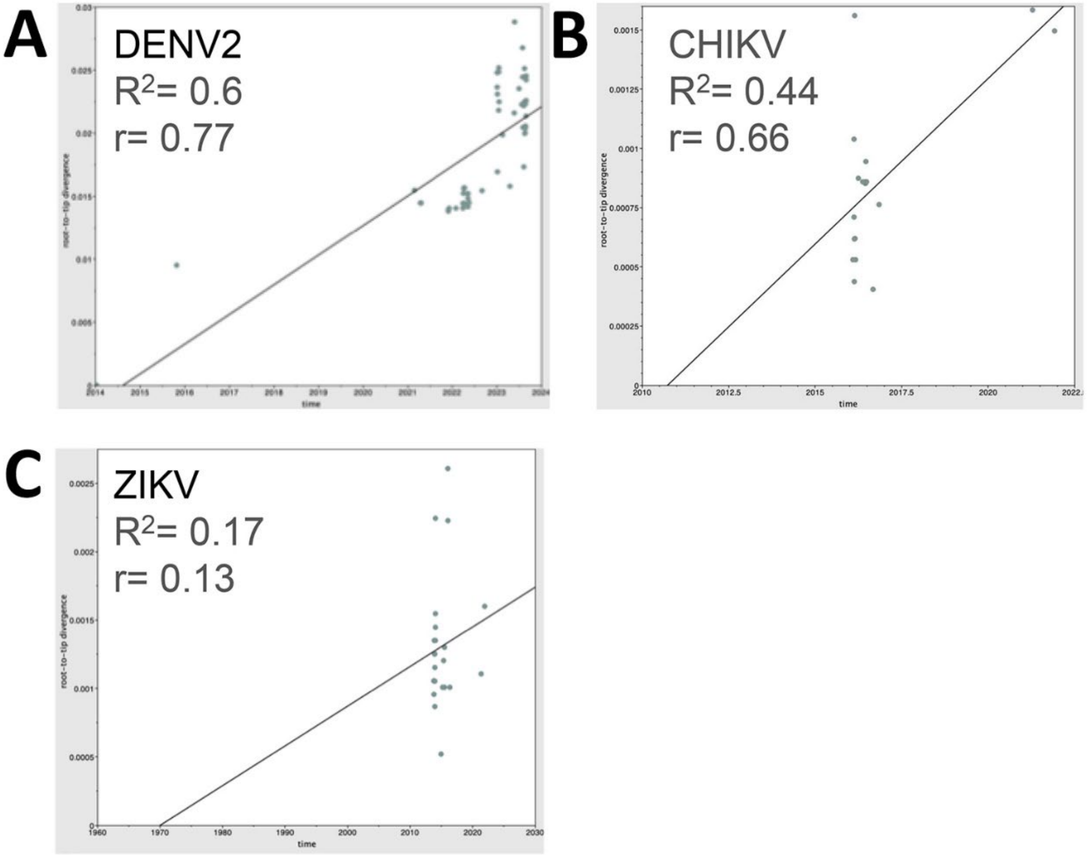

Appendix 1 Figure 1**.** Temporal signal for DENV2, CHIKV and ZIKV. Linear regression of root-to-tip genetic distance within the maximum-likelihood phylogeny of A) DENV2, B) CHIKV and C) ZIKV against sampling time for each taxon. Temporal resolution was assessed using the slope of the regression, with positive slope indicating sufficient temporal signal. R squared (R^2) and correlation coefficient "r" are reported for the dataset.

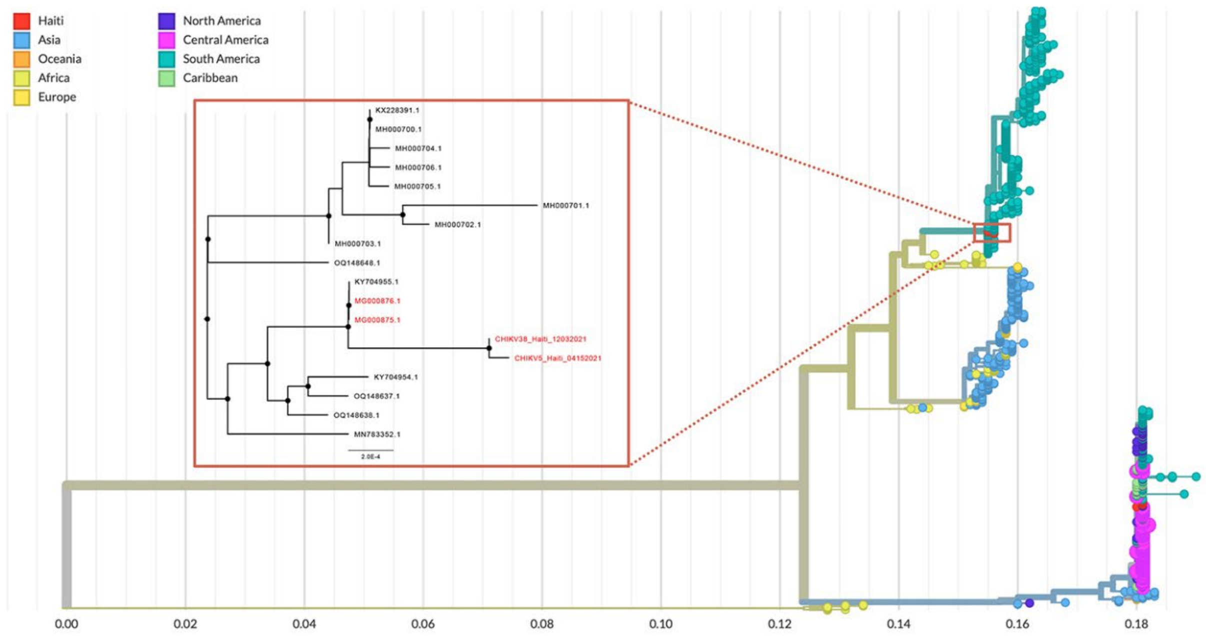

Appendix 1 Figure 2. Global maximum likelihood phylogeny of CHIKV isolates, and phylogeography of the CHIKV Haiti clade II. Maximum-likelihood (ML) phylogeny for all CHIKV isolates available on GenBank were inferred using IQ-TREE v2.3.2. Branch lengths reflect genetic distances, and black circles at each node shows strong statistical support based on ultrafast-bootstrap (BB>90%). The ML tree shows CHIKV lineages colored by region of origin. The zoom shows the Haitian sub-clade, which includes the newly sequenced Haitian isolates.

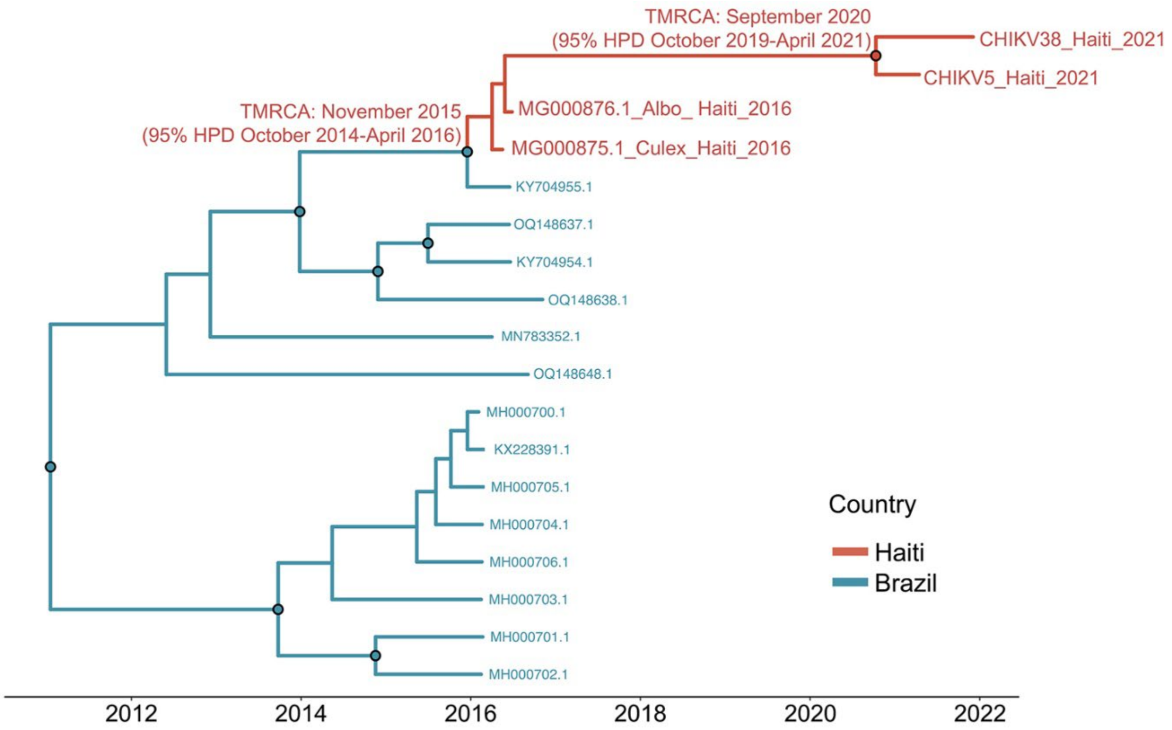

Appendix 1 Figure 3. Time-scaled phylogenetic maximum clade credibility tree for Haiti sub-clade, which was inferred using the phylogeographic frameworks in BEAST v.1.10.14 and enforcing the Bayesian Skyline demographic prior with an uncorrelated lognormal relaxed clock. The Haitian isolates are connected to CHIKV isolates circulating in Brazil suggesting an introduction of CHIKV into Haiti from Brazil.

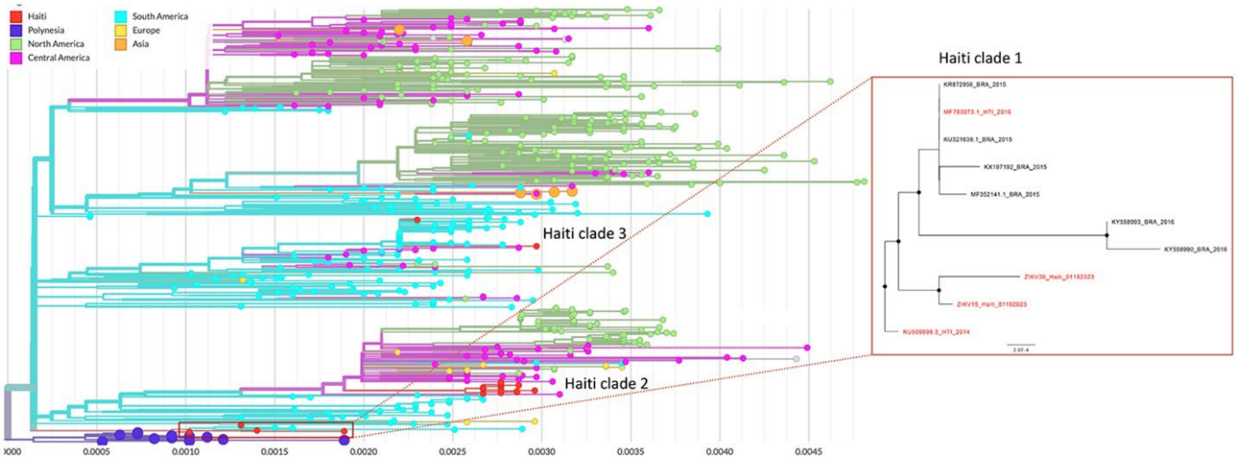

Appendix 1 Figure 4. Global maximum likelihood phylogeny of ZIKV isolates, and phylogeography of ZIKV Haiti-clade-1. M maximum-likelihood (ML) phylogeny for all ZIKV isolates available on GenBank, inferred using IQ-TREE v2.3.2. Branch lengths reflect genetic distances, and black circles at each node

shows strong statistical support based on ultrafast-bootstrap (BB>90%). The ML tree shows clades that contain Haitian sequences: Haiti clades 1, 2 and 3. The zoom shows the clade with new isolates ZIKV36_Haiti_01192023 and ZIKV15_Haiti_01192023, as well as ZIKV isolates from Haiti previously sequenced KU509998.3 and MF783073.1.

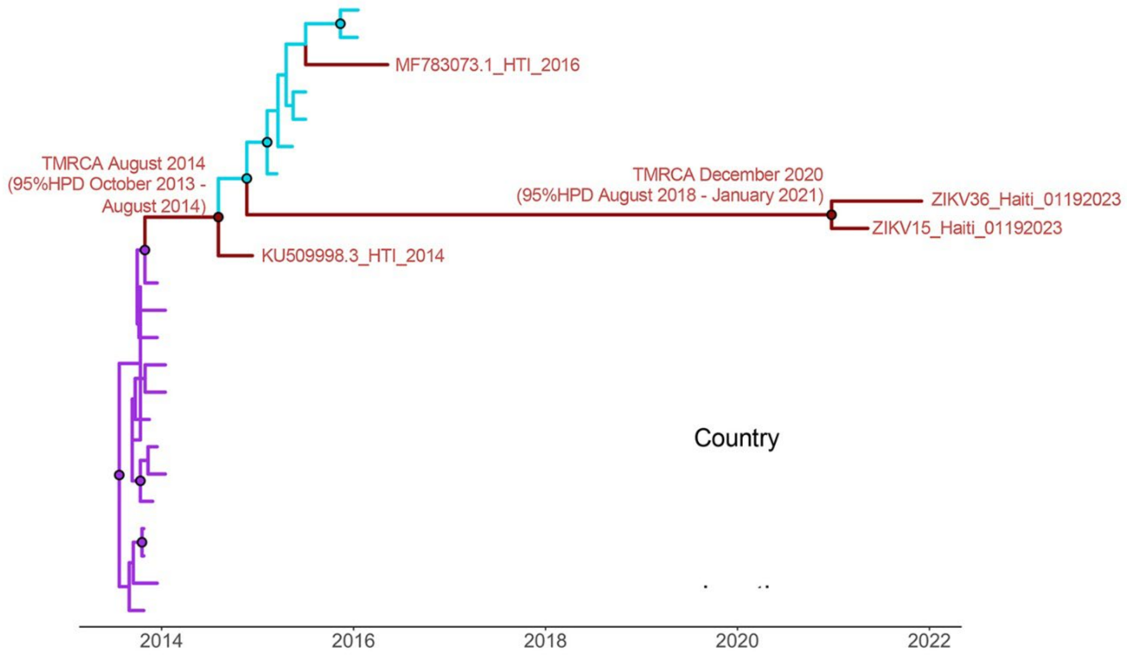

Appendix 1 Figure 5. Time-scaled phylogenetic maximum clade credibility trees for ZIKV that were inferred using the phylogeographic frameworks in BEAST v.1.10.14 and enforcing the Bayesian Skyline demographic prior with an uncorrelated lognormal relaxed clock. Branches are colored based on region/country of origin of the genome, and posterior probability (PP) >0.90 support at each node is shown with a circle that is colored by the ancestral country of origin (Haiti in red, Brazil in cyan, and French Polynesia in purple). The ZIKV isolates from Haiti in the Haiti clade 1 are connected to isolates circulating in French Polynesia and Brazil, suggesting a first introduction from French Polynesia into Haiti with further spread in Brazil and Haiti.
